# Evaluating the use of biobanked urine specimens for human urobiome studies

**DOI:** 10.1128/spectrum.02164-24

**Published:** 2025-10-27

**Authors:** Sromona D. Mukherjee, Ava Adler, Thien Dang, Eric N. Taylor, Gary Curhan, Aaron W. Miller

**Affiliations:** 1 Department of Cardiovascular and Metabolic Sciences, Cleveland Clinic 2569, Cleveland, Ohio, USA; 2 Department of Urology, Glickman Urological and Kidney Institute, Cleveland Clinic 2569, Cleveland, Ohio, USA; 3 Channing Division of Network Medicine, Brigham and Women’s Hospital, Boston, Massachusetts, USA; 4 Division of Nephrology and Transplantation, Maine Medical Center 92602, Portland, Maine, USA; 5 Channing Division of Network Medicine and Renal Division, Brigham and Women’s Hospital, Harvard Medical School 1811 , Boston, Massachusetts, USA; Baylor College of Medicine/Texas Children's Hospital, Houston, Texas, USA

**Keywords:** urine, urobiome, biobanked samples, metagenomics, microbiome, urinary tract microbiome, urology

## Abstract

Case–control studies focused on the urinary tract microbiome, or urobiome, have consistently reported significant associations with disease. However, clinical urobiome studies have typically been small, averaging ~50 patients per study. While these sample sizes are sufficient to detect large effect sizes, they have not been able to differentiate disease phenotypes within a larger disease complex (e.g., different types of kidney stones), which have unique etiological origins. Biobanked urine specimens can help fill this void. However, since these specimens were not collected specifically for urobiome studies, they must be validated before drawing any strong conclusions. The objective of this study was to evaluate microbiome data derived from metagenomic analysis of biobanked urine specimens against the following criteria: (i) level of contaminants; (ii) retention of high-quality DNA; (iii) overgrowth of a few dominant bacteria; and (iv) preservation of sex-specific taxa. A total of 174 samples were assessed from biobanked or freshly collected specimens (*N* = 118 patients total), in addition to multiple positive and negative controls. While there were significant differences in diversity (alpha/beta; *P* < 0.001) based on whether or not samples were biobanked, these differences can largely be explained by study-specific variation. With these criteria, we find that biobanked urine specimens provide similar data to fresh specimens collected using standardized protocols and can be used for clinical urobiome studies.

**IMPORTANCE:**

The urinary tract microbiome, or urobiome, is an emerging field of study that has shown promise as an important contributor to urologic health and disease. However, since this field is relatively new, clinical studies to evaluate the urobiome in the context of urologic disease have been relatively small. The use of biobanked urine specimens would allow for much larger studies to be conducted in a relatively short period of time. However, the use of biobanked urine specimens must first be validated. In this study, we sought to evaluate the use of biobanked urine specimens through multiple metrics, compared to previous studies conducted specifically to assess the impact of the urobiome. Results of our study suggest that biobanked urine specimens produce similar data to urine samples collected under rigorously controlled conditions and can be used in casecontrol studies of urologic conditions.

## INTRODUCTION

A central tenet in urology is that the urinary tract is sterile, except under specific conditions in which the urinary tract is either contaminated through stool or hematogenous spread, leading to infection (1, 2). However, with the advancement of new molecular and culture-based techniques in microbiome research, the sterility of the urinary tract has been called into question, and numerous studies have found that this area normally harbors a resident microflora, including bacteria from the *Lactobacillus, Staphylococcus, Streptococcus,* and *Escherichia* genera, among others, even in the absence of infection, which is termed the “urobiome” (3–13). With the discovery that the urinary tract harbors a resident microflora even in the absence of infection, a new potential etiological pathway emerged for urologic disorders, which impact over 60% of adults (14). Numerous case–control studies have been conducted focused on the urobiome and have consistently found that the urobiome is associated with multiple urologic conditions (3, 15, 16). Importantly, the urobiome is considered a low microbial biomass area, in contrast to the high microbial biomass intestinal microflora (17). As such, urobiome studies must take extra precautions to prevent, assess, and eliminate contamination. Additionally, low microbial biomass areas, by definition, exhibit greater stochasticity in the microbial signatures detected (18). These recognitions have spurred efforts to establish rigorous standards for conducting clinical urobiome studies, aiming to ensure the reliability and reproducibility of findings (19, 20). Standards encompass multiple facets of research methodology that include sample collection, storage, DNA extraction, and analytical approaches. Given the infancy of the urobiome field and the need to develop and validate novel workflows, the clinical urobiome studies published to date have included relatively small numbers of patients, typically ranging from 11 to 210 samples, with some studies lacking controls (Table 1). While these numbers are sufficient to detect large effect sizes, they have not yet been able to differentiate between disease phenotypes within a larger disease complex. For instance, multiple clinical studies have found an association between the urobiome and kidney stone formation (21). However, the question of whether there are microbial signatures unique to different kinds of kidney stones (i.e., calcium oxalate, calcium phosphate, and uric acid) remains unanswered. Biobanked urine samples can potentially be used to increase sample sizes and address more nuanced questions about the mechanistic origins of diseases that can take years to develop, such as kidney stones, focused on the urobiome. However, the question remains as to whether urobiome data obtained from biobanked urine specimens adequately represent the complexity of the urobiome landscape.

**TABLE 1:** Case–control urobiome studies with the number of patients in each cohort^*a*^

Urologic disorder	No. of cases	No. of controls	Reference
Benign prostatic hyperplasia	210	265	(22)
Benign prostatic hyperplasia	41	N/A^*b*^	(23)
Benign prostatic hyperplasia	29	9	(24)
Bladder cancer	48	50	(25)
Bladder cancer	34	29	(26)
Bladder cancer	41	N/A	(27)
Bladder cancer	62	19	(28)
Bladder cancer treatment	11	N/A	(29)
Bladder cancer treatment response	24	23	(30)
Bladder cancer treatment response	42	26	(31)
Chronic kidney disease	46	N/A	(32)
Chronic kidney disease	132	44	(33)
Dialysis	50	50	(34)
Incontinence	159	150	(35)
Kidney stones	24	43	(7)
Kidney stones	50	50	(36)
Kidney stones	30	30	(37)
Kidney stones	16	16	(38)
Kidney stones	52	N/A	(11)
Overactive bladder	21	12	(39)
Overactive bladder	55	18	(40)
Overactive bladder treatment	50	47	(41)
Systemic lupus erythematosus	50	50	(42)
Urethral stricture disease	16	6	(6)
**Average**	**53.7**	**43.4**	

^*a*^ The bolded terms indicate the average number of patients in the case or control groups.

^*b*^ "N/A" means the study did not include healthy patient controls.

To address the question about the utility of biobanked urine specimens for urobiome studies, our objectives were to evaluate the use of biobanked urine specimens from two established biobanks, compared to clinical urobiome data from two recent clinical urobiome studies conducted before and after the implementation of rigorous technical standards (6, 7). To determine the reliability of biobanked samples for urobiome studies, we evaluated microbiome data against the following criteria: (i) level of contaminants; (ii) retention of high-quality DNA; (iii) overgrowth of a few dominant bacteria; and (iv) preservation of sex-specific taxa. By examining urobiome profiles across different sample sources and collected under different protocols, we aim to understand the degree to which biobanked urine specimens capture the nuances of the urobiome. This comparative analysis will shed light on the utility and reliability of biobanked samples for urobiome research, potentially expanding the scope of investigations into urologic health and disease.

## RESULTS

**Fig 1:**
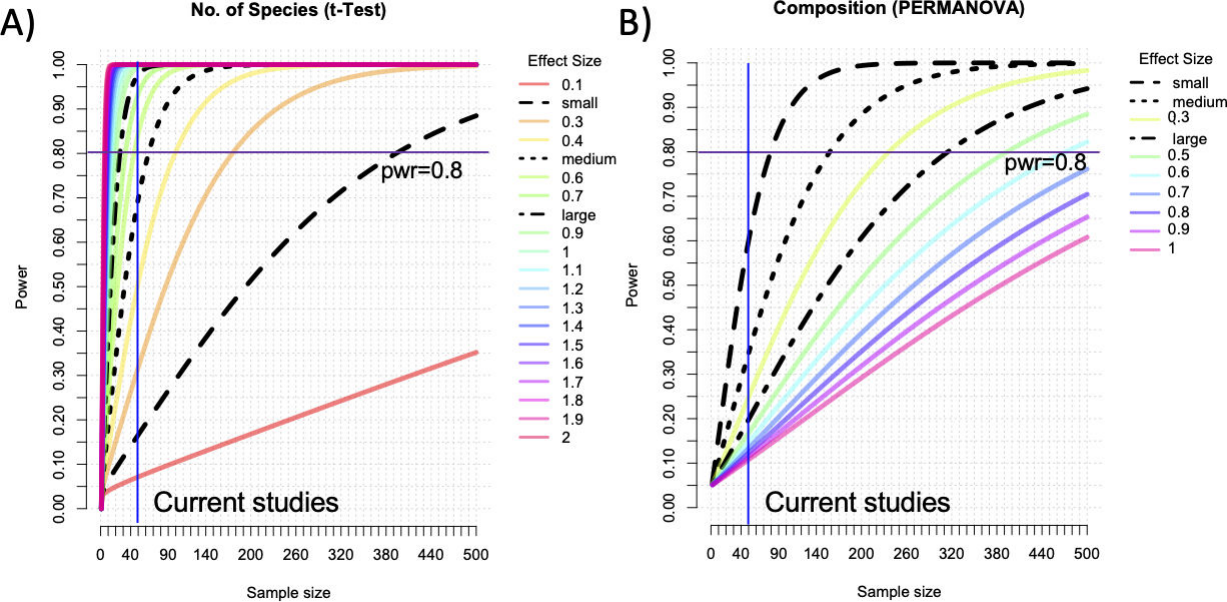
Power analysis. Two critical analyses in microbiome studies include the number of species (alpha diversity) and microbiome composition (beta diversity). These figures show the power for the effect sizes for different sample sizes, for alpha (**A**) and beta (**B**) diversity. Shown are the typical sample sizes of current urobiome studies (vertical blue line), based on averages in Table 1.

To assess the statistical power of previous studies (Table 1), we conducted a power analysis for *t*-tests and PERMANOVA against multiple effect and sample sizes, along with multiple power levels (Fig. 1). Results reveal that with 50 patients per comparative group, the alpha diversity metrics reach a statistical power of 80% for medium effect sizes (0.5; Fig. 1A) while beta diversity metrics only reach a power of 80% at large effect sizes (0.4; Fig. 1B). To achieve a power of 80% at small effect sizes, needed to differentiate disease phenotypes within a larger disease complex or other sub-categorical multivariate analyses, approximately 400 patient samples per group are needed for both alpha and beta diversity analyses (Fig. 1A and B).

### Quality control

**Fig 2:**
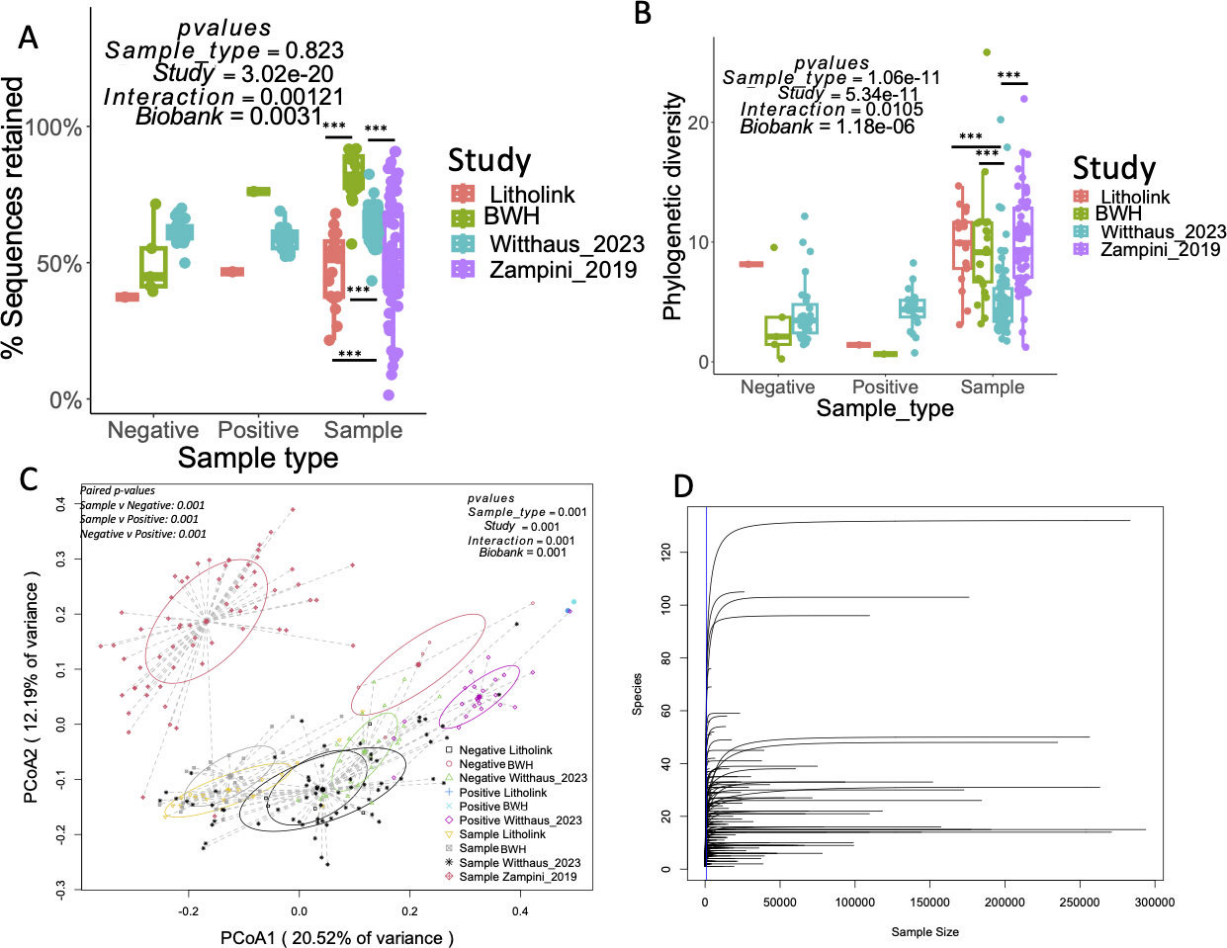
Comparative analyses between urine samples, along with negative and positive controls, by study. (**A**) Proportion of sequences retained after removal of low-quality sequences; (**B**) alpha diversity, provided as phylogenetic diversity; (**C**) beta diversity, provided as a weighted UniFrac analysis; (**D**) rarefaction analysis of decontaminated, biobanked data sets to determine if the sequencing depth was adequate to provide a representative snapshot of the microbiome. ***P-value < 0.001 in Holm’s-corrected paired *t*-tests. *P*-values in one-way or two-way ANOVA or PERMANOVA analyses are shown on plots. Interaction refers to the two-way analysis between the study and sample type.

The retention of sequence reads after removal of low-quality sequences was variable, with no significant differences by sample type (Fig. 2A). However, read retention was significantly associated with biobanking status (98% for freshly collected vs 91% for biobanked; Fig. 2A). However, the variation by study was far more significant. For high-quality, non-decontaminated data, the number of phylogenetic groups detected (alpha diversity) was predictably lowest in negative controls and highest in samples, with the highest diversities in the biobanked samples (6.73 for freshly collected vs 8.49 for biobanked; Fig. 2B). However, the specific study had greater explanatory power for the observed variance than the biobanking status. When considering the microbiome composition (beta diversity), sample types were significantly different from each other, as were each study (Fig. 2C). The specific study (F statistic = 19.992) again was a greater explanatory variable than the biobanking status (F statistic = 12.878). Finally, for biobanked samples, rarefaction analysis dictated that a sequencing depth of 500 sequences would adequately capture the diversity present in urobiome samples. The actual sequencing depth was 34K ± 2K sequences, and all samples had sufficient sequencing depth after decontamination to have adequately captured the diversity present, based on rarefaction analysis (Fig. 2D).

### Comparative analyses by study and biobanking status after decontamination

**Fig 3:**
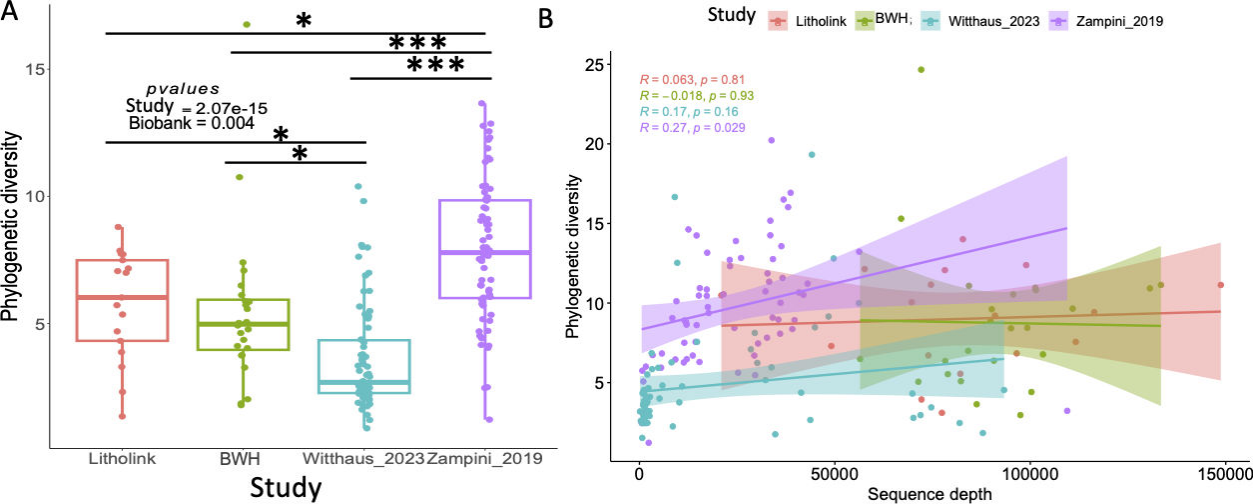
Alpha diversity, quantified as phylogenetic diversity, from decontaminated data. (**A**) Comparative analysis of alpha diversity; (**B**) Pearson correlations between sequence depth and phylogenetic diversity. *P-value < 0.05; ***P-value < 0.001 in Holm’s-corrected paired *t*-tests. *P*-values in one-way ANOVA and correlations are shown on plots.

The phylogenetic alpha diversity of decontaminated urine samples was significantly different by study (Fig. 3A). While there were differences by biobanking status (8.8 for fresh, 7.2 for biobanked), the specific study was a greater explanatory variable. Differences in the phylogenetic diversity from before to after decontamination reflect both the removal of contaminants in samples and the removal of controls that exhibited very low diversity. There was a significant correlation between sequencing depth and alpha diversity only for Zampini_2019 (Fig. 3B).

**Fig 4:**
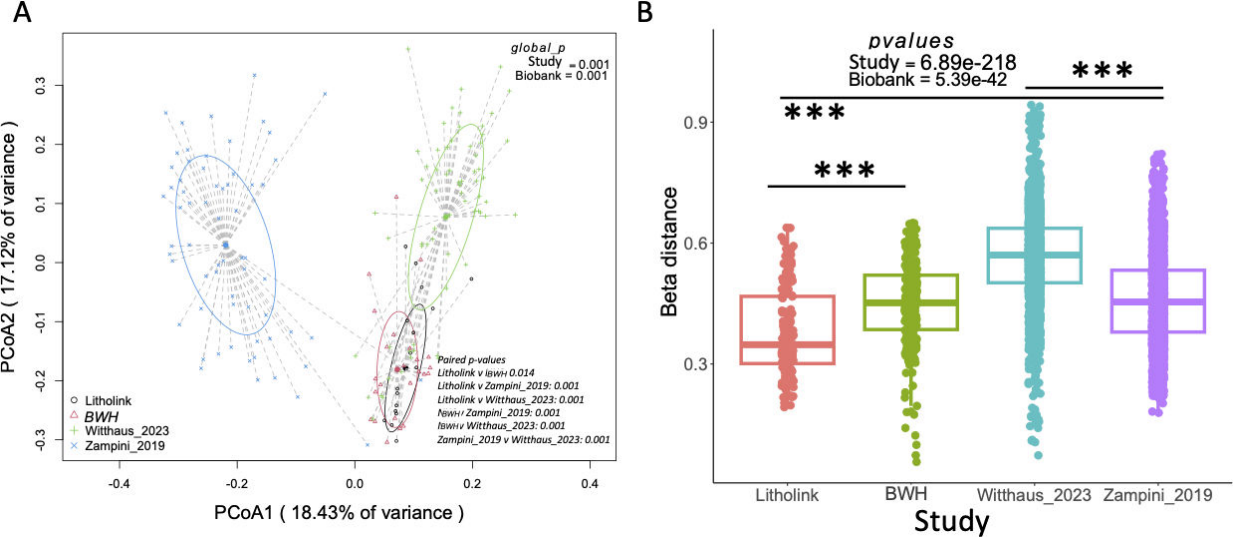
Beta diversity, quantified as a weighted UniFrac dissimilarity, from decontaminated data. (**A**) Comparative analysis of the beta diversity; (**B**) within study variance in the beta diversity, based on all sample:sample pairwise comparisons, compared between studies. ***P-value < 0.001 in Holm’s-corrected paired *t*-tests. *P*-values in one-way and two-way PERMANOVA analyses (**A**) and one-way ANOVA analyses (**B**) are shown on plots.

There was a significant difference in the microbiome composition between each of the studies, quantified as a weighted UniFrac analysis (Fig. 4A). Similar to previous analyses, the specific study (F statistic = 19.622) was a greater explanatory variable than the biobanking status (F statistic = 16.064), and the two datasets derived from biobanked urine specimens were most similar to each other compared to any other pairwise analyses (Fig. 4A). When looking at within-study beta diversity variance, we see that while there were significant differences between studies and biobanking status (Fig. 4B), the specific study was more explanatory of the variance.

**Fig 5:**
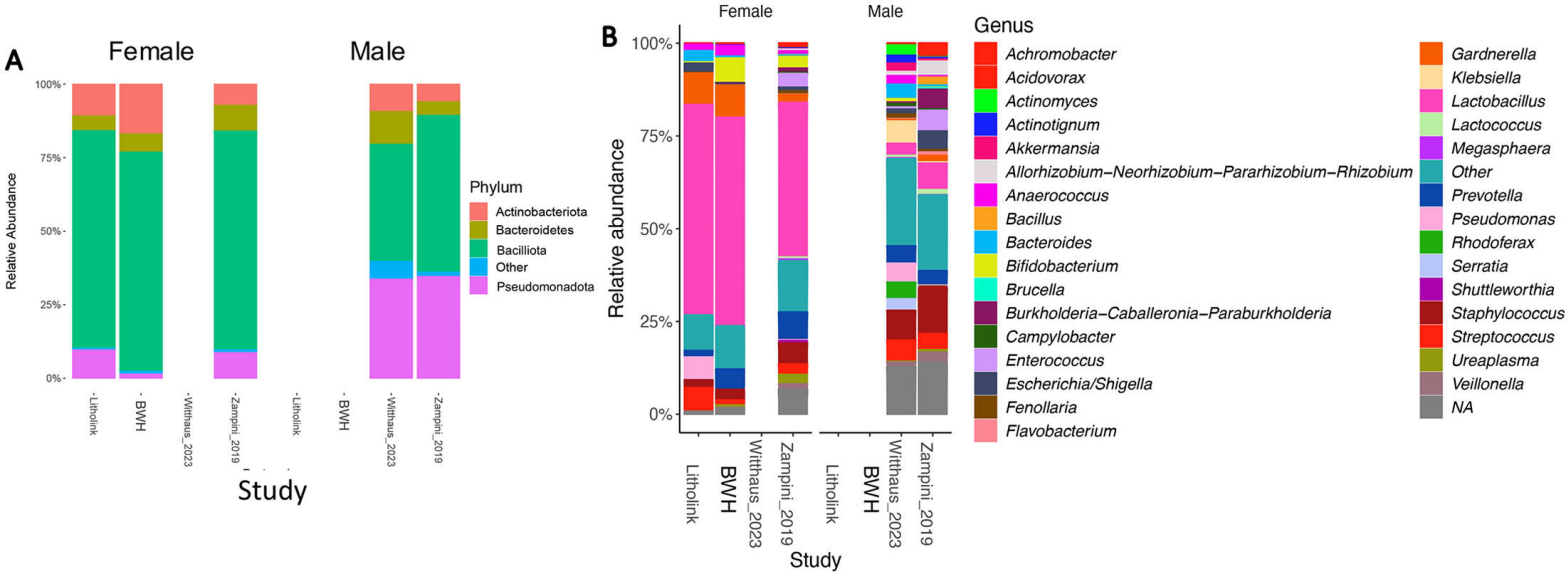
Sex-based taxonomic profiles by study. (**A**) Phylum-level profiles; (**B**) genus-level profiles for the top 33 genera. Values for each taxon were calculated as the proportion of sequences annotated to those taxa, compared to the total number of high-quality, decontaminated sequences.

Taxonomic profiles were generated for each study at the phylum and genus levels, which were similar across studies and were differentiated by patient-reported sex. Importantly, biobanked specimens all came from women, while Whitthaus_2023 all came from men, and Zampini_2019 were mixed men and women. At the phylum level, we see very consistent sex-based profiles, independent of study origination (Fig. 5A). While all data sets and sexes were dominated by Bacilliota, men had higher levels of Pseudomonadota overall. At the genus level, again there were clear sex-based, but not study-based, differences. In particular, the women, independent of the study, were dominated by *Lactobacillus*. The genus profile in men was characterized by a more even distribution of genera, overall (Fig. 5B).

## DISCUSSION

In the last decade, the importance of the urobiome, or resident microorganisms that inhabit the urinary tract, has increasingly been recognized (13, 15–17, 43, 44). Multiple case–control clinical studies have consistently found a urobiome association with multiple urologic conditions (4, 6, 7, 9, 12, 30, 36, 42, 45). However, these early studies generally included low patient numbers, and as a result, the conclusions have been limited (Table 1). The use of biobanked urine specimens can help overcome this limitation, but their use and reliability must first be validated. In the current study, we elucidated the reliability and accuracy of urobiome data derived from biobanked urine specimens compared to those collected using the standardized methodology for clinical urobiome studies.

In the data sets examined here, the alpha diversity, or number of unique phylotypes, was significantly lower in the negatives compared to urine specimens prior to decontamination (Fig. 2B). Furthermore, the microbiome composition was significantly different between negatives and samples (Fig. 2C), independent of the study. As such, data suggest that neither contamination nor technical artifacts, encompassed within negative controls, are important contributors to the microbiome data seen in any of the samples. Importantly, the statistical approach to contaminant/artifact removal used here, provided by Decontam, has previously been shown, in controlled spike-in studies, to reduce known contaminants by >90% with minimal impact on sample bacteria (46). However, one source of contamination not accounted for here is the sample cups in which urine was collected. However, contaminants from sample cups would most likely comprise skin bacteria such as *Cutibacterium, Propionibacterium,* or other genera such as *Bacillus* or *Corynebacterium (30*). We note that *Cutibacterium* and *Propionibacterium* were not found in any data set here, while the *Bacillus* and *Corynebacterium* were only present at very low levels (Fig. 4B). Collectively, data show that the impact of the residual amount of contamination or technical artifacts present in biobanked samples after the decontamination process is negligible. Importantly, however, our findings indicated that the study of origin was the primary determinant of urobiome profiles, with BWH and Litholink biobanked samples clustering more closely together than the freshly collected studies. This suggests that batch effects—arising from differences in patient populations, collection timing, and protocols—significantly influence the microbiome composition. High-throughput 16S rRNA sequencing is particularly sensitive to such batch effects, and even sequencing runs from the same population can yield variation if processed at different time points (47).

One potential concern of using biobanked urine specimens for metagenomic studies is that the DNA will have degraded over time (48). To assess this concern, we quantified the retention of high-quality sequences. If significant DNA degradation occurred in biobanked samples, we would expect lower rates of retention. However, while we do see some differences in sequence retention between the four studies compared and based on whether specimens were banked or not (Fig. 2A), study-specific variation was the greatest explanatory variable. These data indicate that archiving the urine is not an important source of DNA degradation, which would bias the results.

Another potential concern with the use of biobanked urine specimens is that either during collection, before banking, or during storage, some specific, competitive bacteria would overgrow and become dominant within the community, crowding out other slower-growing bacteria. The dominance of specific taxa would produce certain microbial signatures in the data. Specifically, we would see lower alpha diversity overall (49). Second, we would see lower within-study variability in the beta diversity. Finally, taxonomic profiles would be simpler, with fewer taxa overall (50). Looking at the alpha diversity, while we do see differences between studies and biobanking status, these were driven primarily by study-specific variation (Fig. 3A). We do, to some degree, see lower within-study beta diversity variance in biobanked samples vs those freshly collected (Fig. 3B). However, this was likely due to the fact that only women were represented in these studies, which were dominated by *Lactobacillus* compared to the more even distribution of taxa seen in men (Fig. 5). Finally, when looking at the phylum- and genus-level taxonomic profiles, again we do see a lower number of taxa in biobanked vs freshly collected samples (Fig. 5A and B), which was driven by sex-based differences rather than biobanking or study-specific variation, with a dominance of *Lactobacillus* in females, consistent with many previous studies. Collectively, data clearly show that overgrowth by a few dominant taxa did not bias data in biobanked samples.

The final criterion to assess the reliability of biobanked urine specimens is in the preservation of sex-based differences. Past studies have consistently found that self-reported sex is one of the biggest factors that determines the urobiome composition (7, 51, 52). Therefore, sex is an excellent metric to test the reliability. Importantly, we see clear and consistent sex-based consistencies in the taxonomic profiles at both the phylum and genus levels (Fig. 5A and B). However, these data are limited, in that biobanked specimens only included women, while one of the previously published studies only included men. The Zampini_2019 included both sexes. Using the Zampini_2019 data set as the basis for comparison, we clearly see that sex-based differences are preserved in the biobanked samples. While our data do show the expected sex-based differences in biobanked urine specimens, more data are needed for biobanked urine specimens from men.

### Conclusion

Based on our analysis, it is evident that the biobanked urine samples exhibit comparable quality, taxonomic composition, and intra-study variability to studies conducted using standardized protocols for urobiome studies. The observed differences, particularly the higher prevalence of *Lactobacillus* in the female biobanked urine samples, are within the expected range for between-study comparisons. Overall, our findings suggest that biobanked urine samples stored at −80°C or lower even without preservative are well-suited for inclusion in broader epidemiological studies. The data provide a strong rationale for utilizing these samples to investigate larger population trends and associations, thus contributing valuable insights into the field of epidemiology and potentially informing preventive strategies or interventions related to relevant health conditions.

### Limitations

Importantly, the current study was limited, in that only biobanked urine specimens from women were assessed. As such, a similar analysis should also be completed in men. The number of biobanked specimens (*n* = 41) was lower than that of freshly collected specimens (*n* = 133), which could influence statistical outcomes. Additionally, samples were collected under different protocols, which could influence results. Finally, we did not consider any urinary symptoms, comorbidities, or other variables other than patient-reported sex. However, our data indicate that using biobanked specimens is a good avenue for more in-depth analyses to examine the association of these other variables on the urobiome. A larger study including men and larger sample sizes matched by number between fresh and biobanked samples will further add to the robustness of using biobanked samples.

## MATERIALS AND METHODS

### Sample collection and processing

We analyzed stored urine samples from a subset (*N* = 24 samples from 12 patients) of Nurses' Health Study II (NHS II) participants who provided urine in one of two collections. In the first urine collection, more than 1,700 nonpregnant women who were randomly selected based on their reported lifetime history of analgesic use collected a first spot morning urine in the year 2003. The urine was collected without preservatives and shipped overnight on ice to the Brigham and Women’s Hospital (BWH)/Harvard Cohorts Biorepository. The urine was stored in the vapor phase of liquid nitrogen freezers (≤−130°C). We randomly selected urine from 12 participants and analyzed two aliquots from each sample for the current study (denoted as the BWH group) for the present study. For the BWH group, five additional samples comprising sterile water were designated as negative controls, undergoing the entire process from DNA extraction to sequencing, while one sample of commercial DNA was included as a positive. In the second urine collection, more than 2,500 women provided a 24 hour urine sample in the years 2010 and 2011. The urine was collected using the Litholink system with three proprietary additives (a volume marker and two antimicrobial preservatives). After 2 to 3 days at room temperature, an aliquot from ~2,000 participants was frozen at −80°C and stored in a freezer at Litholink. For the present study, we randomly selected 20 women from this collection (denoted as the Litholink group), and 17 had stored urine available, which was used for analyses here (*N* = 17 samples from 17 patients). For the Litholink group, two positives were included, comprising a commercially available mixture of DNA from known bacteria (Zymobiomics). Additionally, four negatives comprising sterile water were included and underwent the entire workflow from DNA extraction through sequencing. Negative controls were included proportionally to the number of samples processed to monitor for potential environmental contamination or technical artifacts. Positive controls were incorporated to confirm successful sequencing runs, particularly in cases where no sample data might be generated. For each biobank study, a single positive control sample was included. The total number of samples, along with positive and negative controls, is presented in Table 2.

**TABLE 2:** Number of samples, replicates, and controls used for the current study

No. of patients	No. of samples	Study	Sample type
22	66	Witthaus_2023	Freshly collected
67	67	Zampini_2019	Freshly collected
12	24	BWH	Biobank
17	17	Litholink	Biobank
NA^*a*^	33	Negatives	Control
NA	25	Positives	Control

^*a*^ "NA" means that the samples were all technical controls (positive or negative), so there were no patient samples.

### Previous clinical studies for comparison

For comparative purposes, we included data from two previous 16S rRNA studies on midstream urine (6, 7), herein denoted as “Zampini_2019” and “Witthaus_2023.” Witthaus_2023 samples were collected, stored, and processed in 2022. This study comprised 66 urine samples (22 patient samples with biological triplicates), with an additional 23 positives and 23 negatives. These samples were handled using standardized protocols. Zampini_2019 samples, collected and processed in 2017, were subjected to processing using published protocols for urine specimens at the time, but before methods were standardized through consensus. This study included a total of 67 urine samples, run as single samples. To ensure that the comparison between fresh and biobanked samples reflected the diversity encountered in clinical settings, both healthy subjects and patients with relevant urological conditions were included in the analysis for freshly collected samples. Biobanked samples were randomly selected. Samples were not stratified by disease status as the primary aim was to evaluate the impact of sample storage conditions on urobiome profiles, rather than to investigate disease-associated microbiome differences.

### DNA extraction and sequencing

DNA extraction from the biobanked urine specimens was conducted following standardized protocols to ensure consistency across all samples. Specifically, 400 µL of pelleted urine was used for semi-automated DNA extraction on a KingFisher Duo Prime system (Thermo Scientific) following the manufacturer’s instructions. The extracted DNA was then submitted to the Microbial Sequencing & Analytics Facility at the Lerner Research Institute (Cleveland, OH) for normalization against DNA content followed by 150-bp paired-end sequencing of the V4 region of the 16S rRNA gene, with primers 515F and 806R on an Illumina MiSeq. All extracted DNA was verified through gel electrophoresis, and DNA concentrations were quantified on a NanoDrop. Concentrations of all samples were >5 ng/µL (range 6.2 ng/µL–42.4 ng/µL).

### Data analysis

Quality control, bimera removal, and taxonomic assignment of amplicon sequence variants (ASV) were completed in dada2 (53). Taxonomic assignment was provided by mapping ASVs to a dereplicated database containing 16S rRNA sequences from the Silva 138 SSURef (54) and NCBI (55) 16S rRNA databases in dada2. Taxa assigned to mitochondria, Eukaryotes, or chloroplasts were removed from all samples prior to subsequent analysis. Additionally, data derived from negative controls were used to remove contaminants from samples using statistical approaches provided by Decontam (56), which effectively remove >90% of contaminants while preserving noncontaminant sequences (46).

From the decontaminated data, the threshold sequencing depths needed to adequately reflect community diversity per sample were calculated through rarefaction analysis in vegan, which we defined as the sequencing depth at which >90% of samples had a slope of <0.01 in the rarefaction analysis (6). For phylogenetic measures of diversity, ASVs were aligned in MSA and arranged into a maximum likelihood phylogeny in phangorn (57) to produce a phylogenetic tree.

From the decontaminated count tables, we calculated the alpha diversity metric phylogenetic diversity in Phyloseq (58). Additionally, the beta diversity was calculated as weighted UniFrac dissimilarities in Vegan (59, 60).

To evaluate the accuracy and reliability of biobanked samples for urobiome research, comparisons were made with the urobiome data examining biobanked (Litholink and BWH) vs freshly collected specimens (Zampini_2019 and Witthaus_2023) as well as between each independently collected set of specimens. Such analysis allowed us to differentiate between the effect of biobanking and study-specific batch effects.

### Statistical analysis

Power analyses were calculated for multiple effect and sample sizes in the R pwr package for *t*-tests and ANOVA. Statistical analyses of sequencing data were performed using appropriate tests, such as ANOVA followed by *post hoc* paired *t*-tests or PERMANOVA. Significance thresholds were adjusted for multiple comparisons where applicable. All statistical analyses were conducted using R, with Holm’s corrected *P*-values < 0.05 considered statistically significant.

### Power analysis

Two common metrics used to compare the microbiome from different experimental or phenotypic groups include the alpha diversity, or number of unique taxonomic groups in a sample, and beta diversity, or microbiome composition, based on the unweighted or weighted presence/absence of taxonomic groups. Alpha diversity metrics are compared statistically with *t*-tests or similar analyses, while beta diversity metrics are compared with PERMANOVA, which is a permutational multivariate ANOVA.

## Data Availability

Sequence reads from the animal study are available at the Sequence Read Archive under accession numbers SRP140641 and SAMN42560143–SAMN42560258.
